# Molar cesarean scar ectopic pregnancy: Report of 2 cases with review of literature

**DOI:** 10.18502/ijrm.v22i2.15714

**Published:** 2024-03-25

**Authors:** Sedigheh Hosseinimousa, Saymaz Navaei, Marzieh Talebian

**Affiliations:** ^1^Department of Obstetrics and Gynecology and Reproductive, Tehran University of Medical Sciences, Shariati Hospital, Tehran, Iran.; ^2^Tehran University of Medical Sciences, Shariati Hospital, Tehran, Iran.; ^3^Department of Obstetrics and Gynecology, Kashan University of Medical Sciences, Shahid Beheshti Hospital, Kashan, Iran.

**Keywords:** Cesarean scar pregnancy, Ectopic pregnancy, Hydatidiform mole, Molar pregnancy.

## Abstract

**Background:**

The occurrence of hydatidiform mole at the cesarean scar site is a rare problem. Few cases have been reported, thus there is not enough information for accurate diagnosis and management of this event.

**Case Presentation:**

Herein, we present 2 cases of an invasive hydatidiform mole embedded in cesarean scar tissue, one presented with occasional hypogastric pain and nausea and another with spotting both with a history of cesarean section. Transvaginal ultrasonography and a considerably high titer of beta-human chorionic gonadotropin blood test suggested the existence of molar pregnancy on the cesarean scar, which was confirmed through histological assessment. In the first case, evacuation of molar pregnancy followed by scar resection at the cesarean scar site led to successful fertility preservation management.

**Conclusion:**

The presence of abdominal pain and unexplained bleeding in a pregnant woman without gestational sac in ultrasonography, strongly suggests ectopic pregnancy. The process of diagnosis should be followed by definitive diagnostic evaluation, including beta-human chorionic gonadotropin titer measurement, ultrasonographic assessment (2 and 3-dimensional), magnetic resonance imaging, diagnostic laparoscopy, and finally biopsy of the lesion.

## 1. Introduction

Gestational trophoblastic diseases contain 2 benign and 5 malignant varieties (including the atypical placental site nodule) (1). The occurrence of hydatidiform mole at the cesarean scar site is a very rare feature; thus, information about the diagnosis and optimal management of cesarean scar molar pregnancy is limited. Early detection may lead to better control of serious consequences such as life-threatening hemorrhage, uterine rupture, and hysterectomy. In pathological assessment, a hydatidiform mole is commonly identified as a hyperplastic placental villous trophoblast cell with interstitial edema, forming blisters of different sizes (2). Although transvaginal ultrasonography can detect hydatidiform mole with an accuracy of 90–95%, the placental trophoblastic tumors present with low specificity ultrasonographic manifestations (3). Color Doppler ultrasound also displays abundant blood flow signals and a low resistance blood flow spectrum. Along with ultrasonic and histological evaluations beta-human chorionic gonadotropin (β-HCG) blood test titer of more than 100,000 IU/L is a major sign of trophoblastic involvement (4). Different treatments are offered for this complication, depending on the clinical condition and the severity and extent of the lesion. However, in many cases, due to the likelihood of severe hemorrhage or uterine rupture, cervical dilation, and curettage, laparoscopic or open lesion resection, and even hysterectomy should be considered as the main approach (1). Because of few current evidences about the disease and the importance of its correct diagnosis, we are presenting 2 cases of molar pregnancy at the site of cesarean scar.

**Figure 1:**
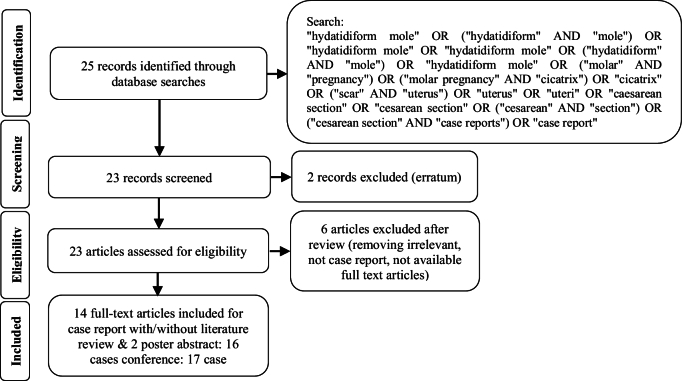
Flow diagram of the screening process.

We performed a comprehensive search through databases containing PubMed and Google Scholar using the above strategy. 23 articles were assessed for eligibility and 6 articles were excluded after review (because they were irrelevant, neither case reports nor full text were available). 14 full-text articles were included and 2 article abstract posters, in total 16 cases remained (Figure 1).

## 2. Case Presentations

This study is a report about 2 cases referred to Shariati hospital, Tehran, Iran.

### Case 1

**Figure 2:**
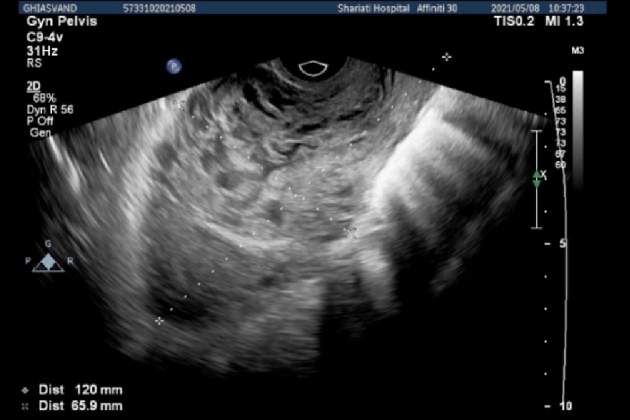
Transvaginal ultrasonography (uterine size = 65 × 120 mm, thin endometrium included abundant cystic areas with increased vascularity around a hyperechoic area in the site of scar).

The first presented case was a 34-yr-old pregnant woman with hypogastric pain and nausea during last week, which intensified in recent days, with tenderness in deep touch of hypogastric region, and a history of cesarean section 4 yr ago. Transvaginal ultrasonography showed that uterine size was 53 × 10 mm (Figure 2), the endometrium was thin including abundant cystic areas with increased vascularity, especially around a hyperechoic area in the site of cesarean section scar with a diameter of 40 × 50 mm. In the color Doppler examination, uterine artery peak systolic velocity was measured at 100 cm/s. The revealed mass favored diagnosing molar debris or pregnancy in the site of cesarean section in the uterus with bulging in the bladder.

**Figure 3:**
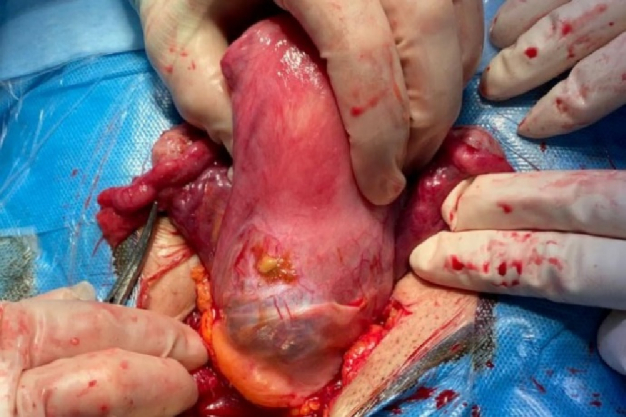
Hydatidiform mole on the cesarean scar.

**Figure 4:**
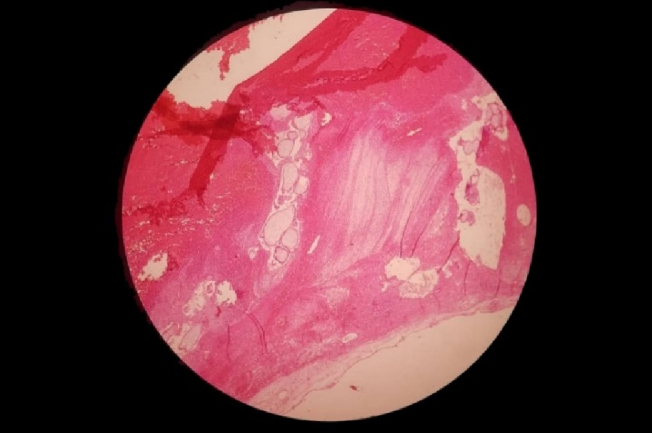
Molar villi in direct contact with myometrium and adjacent to the serosal layer suggesting invasive mole.

The thickness of the myometrium in the area of the cesarean section was reduced to a maximum of 3 mm. In the initial laboratory assessment, the β-HCG titer was 27633 IU/L, and the thyroid stimulating hormone was 3.5 mg/dl. In this regard, suction of molar pregnancy was performed. The patient underwent laparotomy, and a 4 cm mass was removed from the previous cesarean section scar (Figure 3). The pathology report demonstrated the molar villi were in close contact with the myometrium and adjacent to the serosal layer, suggesting an invasive mole (Figure 4). Postoperatively, the β-HCG blood titer was significantly reduced to 1353 mlU/ml.

### Case 2

The second case was a 40-yr-old pregnant woman with spotting during the last month. The estimated gestational age according to the last menstrual period was 8 wk. She had 2 previous cesarean sections at 18 and 9 yr ago. In the physical examination, the cervix was normal. The initial β-HCG titer was 225000 IU/L, and TSH was 1.2 mg/dl.

**Figure 5:**
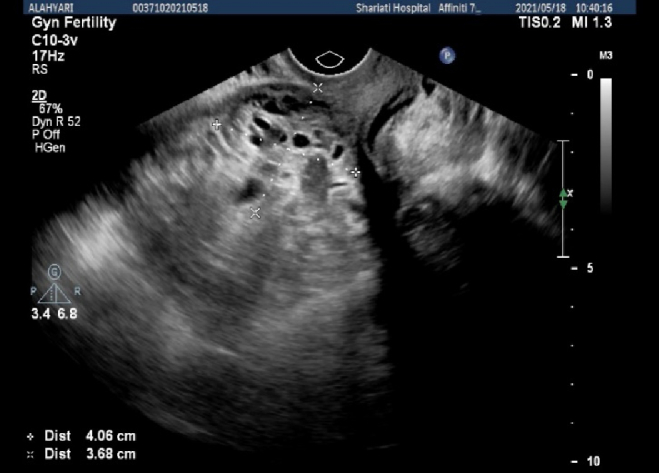
Transvaginal ultrasonography (a heterogeneous thick area containing cystic changes with a size of 59 × 74 mm).

Transvaginal ultrasonography showed the uterine with a size of 58 × 114 mm, a heterogeneous thick area sized 59 × 74 mm with cystic formation and uterine artery systolic peak velocity was 20 cm/s through the Doppler assessment (Figure 5). Observed signs led to the detection of ectopic molar pregnancy on the past caesarian section scar. Hysterectomy and other treatment alternatives, risks, and benefits were discussed; then the patient preferred suction and hysterotomy. She underwent suction, and the pregnancy tissue was evacuated. However, because of severe hemorrhage, immediate laparotomy was performed, and resection of the cesarean scar tissue was done, so bleeding was controlled successfully.

**Figure 6:**
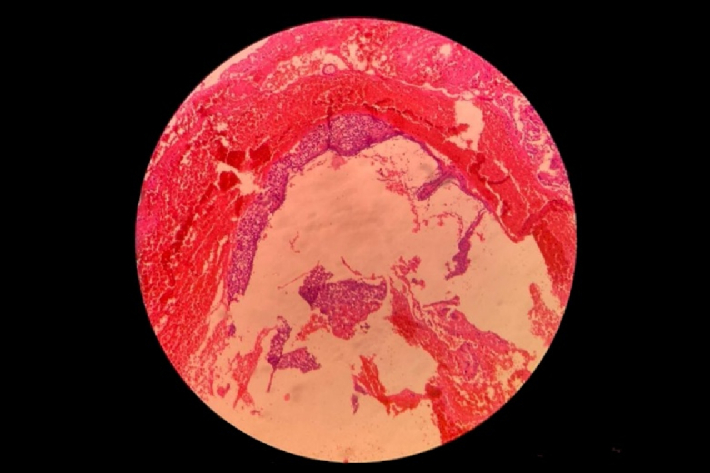
Chorionic villi with abnormal morphology in direct contact with myometrial smooth muscle fiber.

Through the histological assessment (Figure 6), some chorionic villi with abnormal morphology were revealed in direct contact with myometrial smooth muscle fibers, suggesting an invasive hydatidiform mole. According to molar pregnancy follow-up protocols, β-HCG titerage was performed weekly and β-HCG titer decreased to 400 IU/L after 2 months. However, after that β-HCG titer increased to 1200 IU/L; therefore, hysterectomy had to be performed. β-HCG titer decreased to 300 IU/L with no changes after that; therefore, 3 courses of chemotherapy with methotrexate were performed. Follow-up was continued and β-HCG titer decreased to 0 IU/L.

### Ethical considerations

Patients gave informed written consent for writing and publishing this case report. Research Ethics Committees of Shariati hospital, Tehran, Iran has approved the study (Code: IR.TUMS.SHARIATI.REC.1402.116).

## 3. Discussion

The incidence of molar pregnancy is 0.6–8 per 1000 pregnancies (5). Cesarean scar ectopic pregnancy is the rarest feature of ectopic pregnancy and its occurrence rate is between 1:1800 and 1:2216 of pregnancies (1, 6). Therefore, the combined incidence rate is extremely low, with no clear diagnostic or therapeutic approaches. In the last decade, short and long-term complications in mothers and neonates have increased along with the rise of cesarean section (7). Presentations of cesarean scar molar pregnancy are vaginal bleeding, nausea, abdominal pain, and persistent spotting after uterine evacuation (8). Molar pregnancy in the scar of the uterus may have serious complications such as severe hemorrhage and uterine perforation because of the thin myometrium at the site of the scar (9). As the first step in dealing with a suspected case of such complication, special attention should be paid to the ultrasonic diagnostic criteria, including the evidence of “snowflake” and “honeycomb” mixed echoic lesions with high blood supply, presenting “lake” and “dry branch” manifestations in the clinical background with no evidence of uterine or cervical pregnancy, and presenting a defect frequently in the myometrium between the pregnancy sac and the bladder (10). Recent evidence obtained has shown the importance of other diagnostic imaging methods, such as magnetic resonance imaging (MRI). In this regard, using MRI is recommended to clarify the diagnosis of a highly suspected scar pregnancy with abundant blood flow signals indicated by ultrasound. In these cases, MRI shows molar tissue-like tiny cystic lesions, dense enhancement, and intra-tumoral hyper vascularity (3).

Since 2006, 16 cases of hydatidiform mole at the site of cesarean scar have been reported with different presentations, diagnostic methods, and approaches to management. The range of maternal age was 28 to 44 yr. The most common symptom was abnormal vaginal bleeding (13 out of the 17 cases, 76%), other symptoms were lower abdominal pain (7 out of 17, 41%), persistent bleeding after miscarriage (2 out of 17, 11%), without symptom (2 out of 17, 11%), and nausea and unresolving symptoms of pregnancy following a miscarriage. Transvaginal ultrasound and β-HCG titerage are the 2 main methods for diagnosis, and recently MRI has been used to confirm the diagnosis. Treatments varied in different cases, including dilation and curettage, ultrasound-guided surgical evacuation (with or without uterine arterial embolization before the procedure), laparotomy and resection of the mass and uterine repair, recent total laparoscopic management with resection of the mass and uterine repair, and robotic-assisted laparoscopic hysterectomy. Also, chemotherapy with methotrexate and in one case with Etoposide, Methotrexate, Actinomycin D/ Etoposide, Cisplatin were needed (1, 2, 5, 8–20).

A summary of the case reports of molar cesarean scar ectopic pregnancy is shown in table I. Based on the findings in our cases as well as a review of the previously described cases, the presence of abdominal pain and unexplained vaginal bleeding in a pregnant woman with no evidence of a pregnancy sac in ultrasonography and a positive history of cesarean section could be strongly suggestive of cesarean scar ectopic pregnancy. Subsequent diagnostic measures confirming the diagnosis, include titerage of blood β-HCG, ultrasonography, and MRI. Early diagnosis is critical to reduce morbidity.

**Table 1:** Summary of case reports of molar cesarean scar ectopic pregnancy (2006–2023)


**Author, year (Ref)**	**Age (yr)**	**Presentation**	**HCG titer (IU/L)**	**Diagnostic method**	**Treatment**
**Wu ** ***et al.*** **, 2006 (11) **	31	Persistent vaginal spotting	61798	Ultrasound, pathology	Dilation and curettage
**Potdar ** ***et al.*** **, 2010 (12)**	40	Without symptom	106500	Ultrasound, pathology	–
**Jin ** ***et al.*** **, 2011 (10)**	44	Vaginal bleeding, lower abdominal pain	94724	Ultrasound, pathology	Suction curettage
**Biswas ** ***et al.*** **, 2016 (8)**	31	Persistent nausea	15000	Ultrasound, MRI, pathology	Dilation and curettage, then MTX (8 cycles) due to PTD
**Vimercati ** ***et al.*** **, 2016 (13)**	34	Abdominal pain and vaginal bleeding	51547	2D and 3D ultrasound, pathology	Intra gestational sac KCL, local and systemic MTX, resection of the uterine
**Polat ** ***et al.*** **, 2017 (14)**	42	Vaginal bleeding	197525	Ultrasound, pathology	Suction-curettage then multiple dose systemic MTX due to PTD
**Dağdeviren ** ***et al.*** **, 2017 (15)**	34	No symptoms at 5 wk	59705	Ultrasound, pathology	Wedge resection
**Ling ** ***et al*** **., 2018 (1)**	28	Abdominal pain, amenorrhea (48 days), then vaginal bleeding	7894	Ultrasound, MRI, pathology	Uterine arterial embolization, suction evacuation
**Liu ** ***et al*** **., 2020 (16)**	35	Irregular vaginal bleeding > 1 month	193079	Ultrasound, pathology	Bilateral uterine artery embolization and suction evacuation and infusion of 80 mg MTX Intraoperative
**Jiang ** ***et al*** **., 2020 (17)**	35	Vaginal bleeding	1515540	Ultrasound, MRI, pathology	Suction evacuation, uterine arterial embolization, chemotherapy
**Zhou ** ***et al*** **., 2020 (2)**	32	Amenorrhea for 2 months and vaginal bleeding for 15 days	> 225,000	Ultrasound, MRI, diagnostic hysteroscopy	7 rounds of EMA and EP chemotherapy, laparoscopic total hysterectomy BSO, postoperative EMA and EP
**El Miski ** ***et al*** **., 2021 (9)**	30	Acute pelvic pain from 4 hr with 9 wk amenorrhea, severe hemoperitoneum, uterine perforation	Not written	Ultrasound, pathology	Removal of products of conception, uterine repaired
**Cain ** ***et al*** **., 2022 (18)**	38	Vaginal bleeding and abdominal pain	Not written	Ultrasound, MRI	Robotic-assisted laparoscopic hysterectomy
**Kriplani ** ***et al*** **., 2022 (19)**	—	Vaginal bleeding > 1 month	Not written in video article	Did not written in the video article	Medical management failed then total laparoscopic excision
**Hfaiedh ** ***et al.*** **, 2023 (20)**	43	Persistent bleeding after spontaneous miscarriage	80600	Ultrasound, MRI, pathology	Dilation and curettage then intrauterine tamponade balloon by Foley, then emergency hysterectomy and 4 cycles of MTX
**Al-Bataineh ** ***et al.*** **, 2023 (5)**	37	Persistent vaginal discharge with streaks of blood and lower abdominal pain 4 M after a miscarriage	43	Diagnostic laparoscopy, pathology	Laparotomy after diagnostic laparoscopy and removal of the mass
HCG: Human chorionic gonadotropin, MTX: Methotrexate, MRI: Magnetic resonance imaging, PTD: Persistent trophoblastic disease, BSO: Bilateral salpingectomy, EMA: Etoposide, Methotrexate, Actinomycin D, EP: Etoposide, Cisplatin, KCL: Potassium chloride					

##  Data availability

Data supporting the findings of this study are available upon reasonable request from the corresponding author.

##  Author contributions

Concept and design: Sedigheh Hosseinimousa, drafting of the manuscript: Saymaz Navaei, Marzieh Talebian, critical revision of the manuscript for important intellectual content: All authors supervision: Marzieh Talebian.

##  Conflict of Interest

The authors declare that there is no conflict of interest.
